# Adapting Behavioral Interventions for a Changing Public Health Context: A Worked Example of Implementing a Digital Intervention During a Global Pandemic Using Rapid Optimisation Methods

**DOI:** 10.3389/fpubh.2021.668197

**Published:** 2021-04-26

**Authors:** Katherine Morton, Ben Ainsworth, Sascha Miller, Cathy Rice, Jennifer Bostock, James Denison-Day, Lauren Towler, Julia Groot, Michael Moore, Merlin Willcox, Tim Chadborn, Richard Amlot, Natalie Gold, Paul Little, Lucy Yardley

**Affiliations:** ^1^School of Psychology, University of Southampton, Southampton, United Kingdom; ^2^Department of Psychology, University of Bath, Bath, United Kingdom; ^3^NIHR Biomedical Research Centre, Faculty of Medicine, University of Southampton, Southampton, United Kingdom; ^4^Public Contributor, Bristol, United Kingdom; ^5^Public Contributor, London, United Kingdom; ^6^Quality Safety & Outcomes Policy Research Unit, University of Kent & Oxford, Kent, United Kingdom; ^7^Primary Care Population Sciences and Medical Education, University of Southampton, Southampton, United Kingdom; ^8^Public Health England Behavioural Insights, Public Health England, London, United Kingdom; ^9^Behavioural Science Team, Emergency Response Department Science and Technology, Public Health England, London, United Kingdom; ^10^School of Psychological Science, University of Bristol, Bristol, United Kingdom

**Keywords:** intervention - behavioral, optimisation, adaptation, COVID-19, rapid research methods, behavior change

## Abstract

**Background:** A rigorous approach is needed to inform rapid adaptation and optimisation of behavioral interventions in evolving public health contexts, such as the Covid-19 pandemic. This helps ensure that interventions are relevant, persuasive, and feasible while remaining evidence-based. This paper provides a set of iterative methods to rapidly adapt and optimize an intervention during implementation. These methods are demonstrated through the example of optimizing an effective online handwashing intervention called Germ Defense.

**Methods:** Three revised versions of the intervention were rapidly optimized and launched within short timeframes of 1–2 months. Optimisations were informed by: regular stakeholder engagement; emerging scientific evidence, and changing government guidance; rapid qualitative research (telephone think-aloud interviews and open-text surveys), and analyses of usage data. All feedback was rapidly collated, using the Table of Changes method from the Person-Based Approach to prioritize potential optimisations in terms of their likely impact on behavior change. Written feedback from stakeholders on each new iteration of the intervention also informed specific optimisations of the content.

**Results:** Working closely with clinical stakeholders ensured that the intervention was clinically accurate, for example, confirming that information about transmission and exposure was consistent with evidence. Patient and Public Involvement (PPI) contributors identified important clarifications to intervention content, such as whether Covid-19 can be transmitted *via* air as well as surfaces, and ensured that information about difficult behaviors (such as self-isolation) was supportive and feasible. Iterative updates were made in line with emerging evidence, including changes to the information about face-coverings and opening windows. Qualitative research provided insights into barriers to engaging with the intervention and target behaviors, with open-text surveys providing a useful supplement to detailed think-aloud interviews. Usage data helped identify common points of disengagement, which guided decisions about optimisations. The Table of Changes was modified to facilitate rapid collation and prioritization of multiple sources of feedback to inform optimisations. Engagement with PPI informed the optimisation process.

**Conclusions:** Rapid optimisation methods of this kind may in future be used to help improve the speed and efficiency of adaptation, optimization, and implementation of interventions, in line with calls for more rapid, pragmatic health research methods.

## Introduction

Public health interventions can help support protective behavior change during a national crisis (1, 2). However, the rapidly changing context during an ongoing crisis, such as the Covid-19 pandemic, can influence the effects, and delivery of an intervention (3). Context encompasses all circumstances in which an intervention is implemented, and 12 contextual dimensions have been identified including cultural, social and economic, political, and organizational (4). Recent guidance in development for whether and how to adapt behavioral interventions for implementation in different contexts (3, 5, 6) introduces the term “responsive adaptations” to define changes made in response to contextual developments during implementation. The process of adaptation is defined as “intentional modification(s) of an evidence-informed intervention, in order to achieve better fit with a new context” (6). Particular methodological challenges are posed by the need to adapt an evidence-informed intervention to take account of a context which is continually changing, whilst ensuring the intervention maintains an evidence base. Identifying a rigorous yet rapid approach for adapting health interventions is in line with calls for speeding up the pace of health research, to increase its capacity to make relevant and timely impacts on the evolving demands of health services (7). Sharing this approach as a set of methods that can be used more widely could help advance our response to this demand (8).

The Person-Based Approach (PBA) provides a clear process for developing and optimizing interventions with a focus on understanding and accommodating the target users' beliefs about the behavior (9). The PBA has been widely used to optimize interventions prior to Randomized Controlled Trials (RCTs), and could be applied to enable rapid, ongoing adaptation, and optimisation of an intervention during live implementation. Optimisation has been defined as a “deliberate, iterative, and data-driven process to improve a health intervention” (10). The PBA uses in-depth qualitative research to identify barriers to engagement with the intervention and the behavior, and iteratively optimize the intervention to overcome these (11–13). This approach is integrated with theory- and evidence-based behavioral analysis to select an appropriate set of effective behavior change techniques (14). The PBA is used alongside ongoing Patient and Public Involvement, (PPI), which ensures that public contributors are involved throughout the intervention development process and can help address issues arising from the PBA work (15).

This paper aims to provide novel methods to rapidly adapt and optimize an intervention in a rapidly changing public health context. The methods we propose are complementary to the newly developed ADAPT guidance, which provides an overview of the whole intervention adaptation process from first identifying an appropriate intervention to adapt, to considering how to disseminate it (6). Our approach elaborates on the third step of the ADAPT guidance “Plan for and undertake adaptations,” providing specific, detailed methods for conducting and analyzing rigorous, rapid qualitative research to ensure a detailed understanding of the new context, in order to identify necessary adaptations while maintaining an evidence, and theory base for the intervention. We use the example of the “Germ Defense” intervention to demonstrate these methods. Germ Defense is an online intervention which increased handwashing and reduced respiratory tract infection in a large randomized controlled trial (RCT) (16), and is the only proven digital intervention to decrease respiratory disease transmission in the community (17). At the start of the Covid-19 pandemic, Germ Defense was identified as a tool which could be optimized to promote infection control within the home, as the virus causing COVID-19 is transmitted in very similar ways to other respiratory viruses such as the flu virus, which was the target of the original intervention.

## Materials and Equipment

### Intervention

The Germ Defense intervention was first developed to reduce respiratory tract infections and was informed by the Theory of Planned Behavior (18), Protection Motivation Theory (19), and the Common-Sense model of illness (20). The original content was optimized through in-depth qualitative research in line with the Person-Based Approach (9, 21).

A process analysis identified the likely core effective intervention components as: information to raise perceived risk; education about how to perform the behaviors; a goal-setting section with positive feedback when users planned to increase their hand-washing, and encouragement to review the plan when no change was made (22). These components were incorporated into one session which was disseminated to promote cold/flu infection control amongst the general public in 2016–2019 (22, 23).

At the onset of the Covid-19 pandemic, an additional core component (called “Reducing Illness”) was added to Germ Defense which aimed to promote engagement in additional protective behaviors at home when acceptable and appropriate (for example, if symptomatic or vulnerable), including self-isolation, social-distancing, wearing face-coverings, cleaning, and leaving deliveries aside. After a brief introduction to increase awareness of risk from Covid-19 and increase self-efficacy to manage risk, users could choose between the core components of Reducing Illness or Handwashing (24). The intervention is described in full elsewhere (24).

## Methods

### Procedures

The existing digital Germ Defense intervention was rapidly updated in February–March 2020 for the Covid-19 pandemic and first disseminated on 23rd March 2020 in the UK. Subsequently, the intervention has undergone three rounds of further optimisation with revised versions released on 08/05/20, 28/05/20, and 04/09/20, according to the changing context of the Covid-19 pandemic. All optimisations were made whilst the intervention was live in the public domain. Each version of Germ Defense is available from http://archive.germdefence.org/.

**Figure 1:**
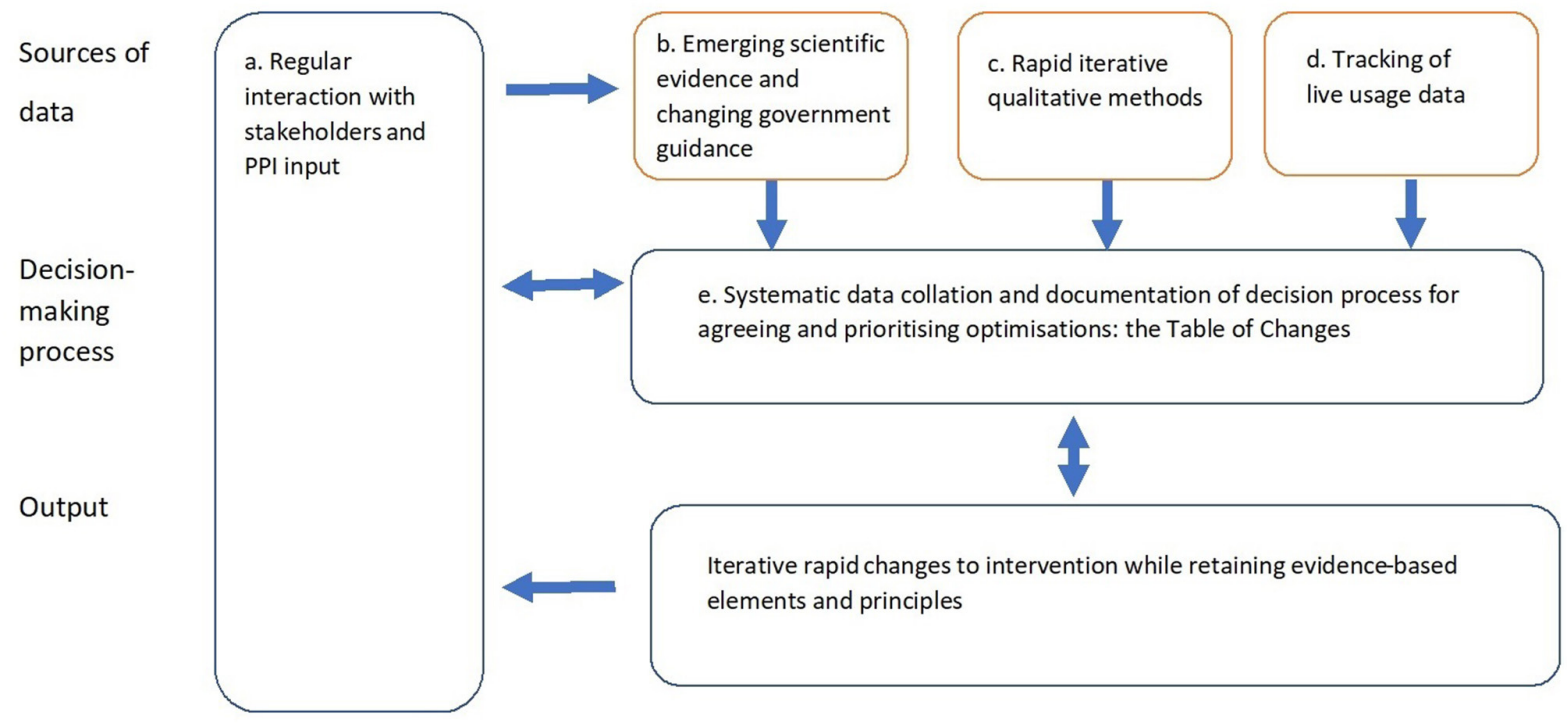
Rapid optimisation methods.

A set of iterative methods were used to rapidly optimize the intervention, as shown in Figure 1.

#### Regular Interaction With Stakeholders and PPI Input

Purpose: To involve a range of experts throughout the rapid optimisation of the intervention to identify high priority changes to ensure the intervention was in line with evidence and persuasive for the target audience.

Methods: Our stakeholders included:

- PPI contributors to provide a public perspective on the intervention optimisation, and help identify and resolve potential issues with acceptability, feasibility, and motivation.
- Clinicians with expertise in infection control to ensure that the intervention was consistent with medical evidence.
- Public Health England partners to ensure that the intervention was consistent with gov.uk recommendations for managing Covid-19.
- Behavior change experts in public health to help ensure the content of the intervention was persuasive and convincing.

Our stakeholder panel was convened at the outset of the project and met weekly *via* online video conferences for 3 months during the most intense phase of intervention optimisation, and then as needed (for example, if there was a change in government guidance or a need to discuss potential changes in response to user feedback). Input from stakeholders was obtained during open discussion at regular meetings and as written feedback using a structured form each time a revised iteration of the intervention was developed (Supplementary Material).

A core intervention development team, comprised of behavior change experts and a computer programmer, were responsible for actioning updates to the intervention that had been agreed with stakeholders, as well as identifying important issues arising from the qualitative research or usage analysis to discuss with stakeholders.

#### Emerging Scientific Evidence and Changing Government Guidance

Purpose: To ensure that the intervention remained consistent with changing government guidance and scientific evidence, which would help people interpret and implement the latest guidance.

Methods: The clinical and Public Health England stakeholders provided essential updates on emerging evidence and changes to government guidance on protective behaviors. In addition, the team received bulletins of the latest evidence around e.g., Covid-19 transmission, and effectiveness of protective behaviors. Key content changes that were needed in response to these updates were discussed with the stakeholder panel, who were also consulted for written feedback on each new version of the intervention.

#### Rapid Iterative Qualitative Research

Purpose: To conduct ongoing in-depth qualitative research with the target population at speed to understand public perceptions about the pandemic and help inform optimisations to the intervention to increase persuasiveness, relevance, and engagement with the protective behaviors.

Methods:

Three methods were used to explore public perceptions of the intervention:

- Qualitative telephone think-aloud interviews (9, 25) were conducted by Health Psychology researchers to gain in-depth understanding of users' perceptions about the behavioral advice on each page of the intervention, as well as their general perceptions and experiences of staying safe during the pandemic. We aimed to speak to a range of people in terms of age, gender, ethnicity, experience with Covid-19, health literacy, and motivation to use Germ Defense and engage in protective behaviors. This was important to ensure that the intervention was persuasive and accessible to as many people as possible. The interviews were analyzed using the Table of Changes, as described further in section **Systematic data collation and documentation of decision process for agreeing and Prioritizing optimisations: the Table of Changes**. This study will be described in full in a separate paper.
- An online survey collated open-text feedback from users of the intervention about what was liked, disliked, should be changed and their experiences of putting the target behaviors into practice. All users of Germ Defense were invited to leave their details if they were interested in participating in research to improve the intervention, and those who provided their email address were subsequently emailed the survey.
- An online PPI activity sought feedback from the People in Health West of England PPI group regarding three alternative front-page designs for the Germ Defense website.

The research was approved by the University of Southampton and University of Bath ethics committees (Registrations 56445 dated 12/05/20, and 20-088 dated 19/03/20).

#### Tracking of Live Usage Data

Purpose: To understand intervention usage and aggregated trends in current and planned adherence to self-reported target behaviors, to help identify possible optimisations.

Methods: The intervention software captured every visit to the website, including which pages the user viewed, and self-reported frequency of current behaviors and behavioral intentions. Users agreed to this when first accessing the website. This enabled us to explore whether the intervention was changing behavioral intentions, and identify the most common points of attrition from the intervention, which provided another source of data for informing optimisations.

#### Systematic Data Collation and Documentation of Decision Process for Agreeing and Prioritizing Optimisations: The Table of Changes

Purpose: To collate stakeholder and user feedback from a wide range of sources, including surveys, emails to the research team from intervention users, stakeholder discussion, and qualitative interviews, and identify important changes required to promote behavior change.

Methods: The Table of Changes is a method promoted as part of the Person-Based Approach (9, 12) for identifying optimisations to an intervention. It facilitates the process of reviewing in-depth qualitative data by collating quotes relating to each aspect of the intervention and encouraging the researcher to review these using a set of criteria to identify why an optimisation might be warranted, and how important this is in terms of achieving engagement and behavior change using the MoSCoW criteria (Must have, Should have, Could have, Would like) (26).

Possible optimisations to the intervention were informed by guiding principles, which described the design objectives for the intervention based on our understanding of barriers to behavior change during the Covid-19 pandemic, and key intervention features to achieve these objectives. These guiding principles helped decide whether an optimisation was in line with the intervention's remit or not.

The following steps were taken to maximize the effectiveness of the Table of Changes in a rapid optimisation context.

- Given the need to rapidly evaluate large volumes of data and feedback from multiple sources, the Table of Changes was set up as an online shared spreadsheet which enabled different team members to input data simultaneously. This helped the core intervention development team rapidly transfer incoming feedback to the Table, and identify possible changes which were high priority (defined as being likely to impact on adherence to the target behaviors (12).
- New columns were incorporated into the Table of Changes to quickly identify which version of the intervention a comment related to (as the intervention went through many minor interim versions between the live versions that were launched), and the status of possible changes i.e., actioned, or outstanding while awaiting more evidence.
- To better inform decision-making about possible optimisations to the intervention, any perceptions about Covid-19 and the protective behaviors which arose during participant interviews were linked back to specific intervention content in the Table of Changes. For example, when a participant discussed their perceived risk of catching the virus, this was linked to the intervention content concerning risk messaging, whilst experiences of wearing a face-covering were linked to the intervention content containing guidance about this protective behavior. This allowed the team to collate general perceptions about the public health crisis alongside intervention-specific feedback initiated during the think-aloud interview, which helped ensure that decisions about how to optimize intervention content took account of broader public perceptions in the current climate.

## Results

These results discuss how each method contributed to the rapid optimisation of the Germ Defense intervention. Specific examples of their application to the Germ Defense intervention are provided as boxed case studies. These optimisations were made to the intervention whilst it was live with over 100,000 users up to 31st October 2020.

### Regular Interaction With Stakeholders and PPI Input

Having a responsive stakeholder panel with representation of members of the public and clinical, behavioral, and public health experts was essential to inform priority optimisations to the intervention. Working closely with PPI contributors flagged up if a message was not acceptable or persuasive, or if the intervention had failed to address important questions. Clinical and public health stakeholders ensured that the advice was in line with medical evidence. The structured form for collating written feedback facilitated the systematic, rapid capture of stakeholder views which could be collated and easily linked to intervention content, and encouraged stakeholders to separate changes they considered essential from lower priority feedback.

Box 1 shows specific examples of rapid high priority stakeholder feedback and how this contributed to the intervention optimisation.

Box 1 **Specific examples of rapid intervention optimisation in response to high priority stakeholder feedback.**

Table 1 shows examples of how stakeholder feedback informed intervention optimisation.

**Table 1:** Examples of how stakeholder feedback informed intervention optimization.

**Stakeholder role**	**Feedback and date received**	**Optimisation**
PPI	Can the virus be caught through the air, as well as picked up from surfaces? (01/05/20)	It was essential that the airborne transmission of the virus was clear in the intervention as a possible route of infection, to ensure that users understood the risk, and the rationale for the protective behaviors. Therefore, the page explaining transmission was modified to clarify that the virus can be caught both from touching contaminated surfaces and by breathing it in through the air. Detail was added about how long the virus can remain in the air.
Clinician	Ensure the message about viral load is consistent with the evidence available on Covid-19 (07/04/20).	Germ Defense originally included a motivational message about the benefit of reducing viral load, to increase perceived control over staying well. The clinicians ensured that this intervention content was still consistent with the evidence available on Covid-19.
Behavior change specialist	The behavior review and goal-setting were important behavior change techniques used in Germ Defense version 1, but asking people to review and plan seven specific behaviors over two separate pages in the handwashing component led to relatively high attrition at this point of the intervention (09/03/20).	The number of behaviors that users were asked to review and plan in each core component was reduced to five behaviors presented on one page, to reduce burden on users. Evidence on transmission routes was used to help design the measures, to select behaviors known to be most important to improve infection control due to different transmission routes.

### Emerging Evidence and Changing Government Guidance

Remaining up to date with emerging evidence and changing government guidance during a national crisis was essential for ensuring that the intervention remained not only relevant but also persuasive. Newly emerging evidence was raised by stakeholders at regular meetings, and plans for how to incorporate this evidence into the intervention were discussed. This included ensuring that the guidance on face-coverings and social distancing was consistent with Public Health England (PHE) guidance, which was essential for agreement by PHE to signpost to Germ Defense in national guidance. Using software which enabled changes to live website content was critical for ensuring these changes could be rapidly implemented.

Box 2 shows an example of rapid iterative optimisation to the Germ Defense content in line with emerging evidence (27, 28).

Box 2 **Example of rapid intervention optimisation in response to emerging evidence or changing government guidance.**

When launched in March 2020, the intervention briefly mentioned opening windows as a supplement to self-isolation:

“Can you arrange for one room in your home to be yours, and that you spend as much time there as you can? This could include eating or sleeping. Opening windows and limiting the amount of time you are in a room with others can also help.” (Germ Defense version 3, released March 2020).

By April 2020, further evidence had emerged about the importance of opening windows for improving infection control (24), and the stakeholder group agreed that this protective behavior needed to be promoted in its own right. In the next iteration of Germ Defense, opening windows was suggested as an effective protective behavior independently from self-isolation, and a rationale was added to increase positive beliefs about the consequences of opening windows:

“Opening windows stops the virus collecting in the air”. (Germ Defense version 4, released May 2020).

At the end of July 2020 the World Health Organization released guidance emphasising the importance of ventilation to introduce fresh air and help reduce airborne transmission (25), and the stakeholder group agreed that given the growing evidence, further, content needed adding to the intervention to increase motivation to open windows. Our PPI and behavior change specialists agreed that raising risk perceptions about the airborne transmission of Covid-19 could help increase motivation to open windows more often. The stakeholder group also identified that opening windows would be more challenging in the upcoming colder months, when people might struggle to heat their home. Working closely with our PPI contributors, the following messages were agreed for Germ Defense Version 5:

Message to raise perceived risk: “Coronavirus can stay in the air for up to 2 h indoors after being breathed out. This means that the virus may stay in the air in your home even after an infected person has left the room.”

Message to increase self-efficacy to open windows when needed without risking too much heat loss: “Opening windows often is an easy way to stop the virus collecting in the air. If it is cold outside, you could open a window in one room if you're planning to spend time there with someone else you live with, or a visitor. Shutting the door to the rest of your home will reduce the amount of heat lost.

You could choose a room that is easier to heat up after or that you don't spend much time in, such as the kitchen.” (Germ Defense Version 5, released September 2020).

### Rapid Iterative Qualitative Methods

The qualitative think-aloud interviews, open-text surveys and PPI activities provided essential insights into barriers to adhering to the target behaviors. An example of how PPI consultation informed optimisation is provided in Box 3, but it is beyond the scope of this paper to present detailed findings from the qualitative methods, which will be published separately. Instead this paper provides an overview of the role of these methods in collecting qualitative data to feed into decision making.

Box 3 **PPI survey to inform optimisation of the intervention front page.**

We anticipated greater usage of the intervention on mobile phones in the Covid-19 pandemic, rather than on larger devices such as PCs for which the intervention format had originally been designed, 12 years previously. Therefore, we sought to optimize the front page of Germ Defense to clearly pull out the key messages for new users and minimize the need to scroll when reading on a smaller screen.

Two alternative shorter versions of the front page were produced by the core team and PPI stakeholders, one a similar design to the original but with less text, and the other a more colorful version with even less text to read. An online survey was sent to the PPI group “People in Research West of England” to gain rapid feedback on the current front page and the two new possible designs. The survey explained the rationale for redesigning the front page, and asked respondents to select which of the three options they liked best. Open-text boxes captured what they liked, and whether they felt anything should be changed.

Fifty-nine responses were received from 440 invites (13%). The number of people choosing each of the three options is shown in Table 2.

**Table 2:** *N* (%) of sample selecting each option as their preferred version of the front page.

**Front page version**	***n* (%)**
Option 1 (existing version)	5 (8%)
Option 2	10 (17%)
Option 3	44 (75%)

The majority of the sample preferred option 3, and open-text feedback indicated that this version of the front page was regarded as simple and visually engaging. The feedback was used to further optimize the front page and a revised version was launched in September 2020.

Usage data did not suggest any effect from the change, with the attrition rate (proportion of users closing the website) from the front page standing at 27% (36,163/135,492 sessions) before the front page was updated, and 26% after (6,778/26,026 sessions). However, there are limitations in interpreting usage data from before and after a change during real-world implementation, as contextual factors are continually changing.

Recruiting research participants from users of the online intervention was a successful approach: from April to October, approximately 1.7% of users (668/38,945 who reached the end of a core component) registered their interest in taking part in research to improve the intervention. However, initial think-aloud interviews conducted with seven participants who had volunteered *via* the intervention suggested these participants were generally very knowledgeable and motivated to adhere to protective behaviors. In addition, most participants described having the space in their home to self-isolate if needed. Therefore, further think-aloud interviews were conducted with six participants recruited from community sampling *via* social media. This enabled us to speak to people who had not actively sought out the intervention, and thereby understand some of the barriers to the target behaviors amongst a less motivated sample.

In terms of methods of data collection, the think-aloud interviews provided in-depth insights into perceptions of the intervention and helped inform optimisations to overcome behavioral barriers. The online survey responses (*n* = 125) complemented the interviews by providing feedback from a broader range of people. Importantly, we found that the open-ended survey responses generated similar themes to the think-aloud interviews in terms of the barriers and facilitators to engaging in protective behaviors (29), which helped to confirm that we were successfully identifying common concerns within the target population despite only having resources to conduct limited think-aloud interviews.

Rapid consultation with PPI was used to seek feedback on how best to optimize the intervention from an informed group who regularly engage with health research teams. Box 3 describes how a PPI survey informed decisions about the content and design of the front page.

### Tracking of Live Usage Data

Comparing self-reported current behavioral frequency and behavioral intentions after using the intervention helped confirm that the intervention was successfully increasing people's intentions to perform protective behaviors more frequently (24). Self-reported goal-setting data also provided insights into which of the target behaviors users intended to perform most frequently (cleaning and putting things aside), and which they intended to do least often (wearing face-coverings at home) (24), which helped inform ongoing optimisations to the intervention ^1^.

Usage data were also used to understand at which points of the intervention people were most likely to disengage, to inform potential optimisations for promoting engagement. Box 4 provides an illustration of how these live usage data were used to inform rapid optimisations.

Box 4 **Usage data on attrition from the intervention.**

The usage data revealed that one of the most common attrition points was the page where users were asked to input their current frequency of performing five target behaviors. At the time, users were only given a brief introduction to explain what to do:

“Think back over the past week and circle the answers that best describe your situation. Please click on one circle for each activity.”

The core intervention development team suggested that this activity may have come as a surprise, being the first interactive activity after reading several pages of guidance, and the stakeholders agreed that adding a short rationale to explain why users were being asked to complete this self-monitoring activity might help to reduce attrition at this point. We worked closely with our PPI contributors to ensure that the message was persuasive, as follows:

“The questions below are about what you already do at home. Answering them takes a little bit of effort, but other people have found this very helpful.”

However, there was no evidence for any impact of this change as attrition rate on this page was 16.95% (8,057/47,523) before the explanation was added and 18.77% (3,466/18,465) afterwards. While these attrition rates are both relatively low for a public health intervention, qualitative research might help understand how to further increase engagement with self-reporting behavior.

### Systematic Data Collation and Documentation of Decision Process for Agreeing and Prioritizing Optimisations: The Table of Changes

The Table of Changes enabled clear collation and tracking of all emerging evidence, government guidance, qualitative research findings, and live usage data for the Germ Defense intervention, as well as providing a record of the decisions made about which changes to make. Box 5 shows an excerpt from the Table of Changes which was modified for this rapid optimisation project.

Box 5 **Table of Changes excerpt.**

Table 3 shows an excerpt from the Table of Changes which was used to track all sources of feedback on the Germ Defense intervention, proposed optimizations to the intervention, and action taken.

**Table 3:** Excerpt from the Table of Changes to show systematic collation of intervention feedback, possible changes, and action taken.

**Intervention page**	**Website version**	**Source**	**Date received**	**Negative comments**	**Positive comments**	**Neutral comments**	**Possible Change**	**Reason for change**	**MoScoW**	**Implementation stage**
Protect yourself1	3	Stakeholder meeting: PPI contributor	01/05/20	The airborne nature of the virus is unclear – can it only be picked up from touching surfaces or can it also be breathed in through the air?			Emphasize on this page that the virus can be breathed in as well as picked up from touching contaminated surfaces, to increase perceived risk and explain the rationale for the protective behaviors, such as face-coverings.	Important	Must have	The change was discussed with stakeholders and clinicians confirmed the virus can be transmitted *via* aerosols. Clearer wording was agreed for the intervention to convey this important route of transmission. Implemented Version 4.
Protect yourself3	4	Interview: P3	12/06/20	“I don't know whether anti-bac wipes are enough or, you know, just the ones you buy, or whether just diluted bleach is okay, or whether there's something better that I should be using.”			Add information to explain how to make a diluted bleach solution, and how this compares with antibacterial spray for effectiveness.	Not changed	Could have	No change made: Agreed at stakeholder meeting this was too specific and detailed for the general population. The optional extra session on Reducing Illness already includes information about the type of disinfectant to use.

## Discussion

This paper describes a rigorous PPI approach to understanding how best to adapt and optimize intervention content to meet users' needs and achieve behavioral outcomes in a changing public health context. Whereas, in more traditional intervention development using the PBA and PPI we might allow 6–9 months for optimizing the intervention (12), in this case we optimized and launched revised versions of the intervention within just 1–2 months.

The following key methods are proposed for teams undertaking rapid adaptation and optimisation of an intervention in a changing public health context. The scale of each activity can be adapted according to the project's time and resources, but sufficient feedback is needed to ensure that optimisation remains grounded in evidence and the perspectives of the target population:

- Regular stakeholder input *via* meetings and written feedback helps ensure that the intervention is consistent with evidence and guidance.
- Think-aloud interviews provide an in-depth understanding of the underlying beliefs and rationale that inform people's decisions about changing their behavior, which can help identify important optimisations. Open-text surveys can supplement interviews to rapidly collect data on common issues with the intervention and target behaviors in a wider population.
- PPI contributions were critical for informing decisions about how best to optimize the intervention to address issues raised by the qualitative research, usage data, or emerging evidence.
- The Table of Changes remains a useful tool in a rapid adaptation context, and can be modified to suit a particular project's needs.

Additional considerations are suggested for how researchers might undertake these rapid optimisation activities:

- Inviting users of the intervention to participate in qualitative research to inform optimisation worked well. In this case, website users could click on a link to read more about how to register their interest in participating, but non-digital interventions could also use this approach by including details of an email address or sign-up page which people can access if they want to contribute to intervention optimisation.
- Recruiting participants for qualitative research from a community sample as well as intervention users can help ensure a wider range of views on the intervention and target behaviors are captured. This can be more challenging during rapid, remote research as effective methods for recruiting less motivated participants often involve attending community events, and building rapport with the target population (30), but in this study, posts within community Facebook groups tended to be well-responded to. The need for reaching less motivated participants might depend on intended dissemination routes and target reach of the intervention.
- For digital interventions, user feedback and usage data can provide valuable insights into users' perspectives on performing the target behaviors, as well as common points of disengagement. Collecting demographic data from users could further enrich these insights by providing information about who is using the intervention and for whom it is most effective. However, decisions about collecting user demographics during rapid implementation of an intervention may need to be informed by a balance between seeking optimal evidence of effectiveness and engagement, and the need to minimize barriers to engagement with an intervention in a real-world setting.

The following sections consider wider implications from this paper in terms of intervention adaptation and rapid research.

### Breadth and Depth of Stakeholder Involvement

The importance of a carefully selected stakeholder team is consistent with the new ADAPT guidance, and has been recognized as essential for rapid-learning research systems (7). We support the value of working with stakeholders from the four groups identified by ADAPT: public contributors from the target population, researchers familiar with intervention optimisation, stakeholders involved in intervention delivery, and those responsible for wider implementation. We also incorporated a fifth group of stakeholders who we term “experts,” in this case clinical and public health experts, who were able to provide advice on the behavioral guidance and emerging evidence. This set of methods outlines an approach for PPI and stakeholder involvement which is compatible with rapid research; discussing emerging issues with stakeholders, working together to identify optimisations which were then implemented by a core intervention development team, and subsequently collating written feedback on the updated intervention from across stakeholders. This clear strategy for stakeholder involvement helped ensure efficiency, which has been identified as a potential pitfall if stakeholder involvement is not carefully planned (31).

### Quality of Rapid Research

Rapid research can inform responses to public health emergencies in local populations and help understand changes in public perceptions during a crisis, but maintaining quality in recruitment methods, data collection and analysis during rapid research can be challenging (32). Transparency in reporting the contributions and limitations of rapid research methods is important for improving quality (32).

The present example showed how recruiting interview participants from volunteers who had actively sought out the intervention and then chosen to register their interest in research could lead to limited diversity in sampling such that only the views of highly motivated, highly health literate individuals were represented. Adopting more than one approach to recruiting participants was important to ensure greater variation in people's perceptions about the intervention and target behaviors. Secondly, in terms of data collection, in-depth think-aloud interviews were successfully conducted remotely rather than face-to-face without any apparent loss of depth or rapport. These were ordinary telephone interviews in which the interviewer followed the participant through the intervention by asking them to say when they were moving onto the next page, which is an approach that could be used for both digital and non-digital interventions. While this was necessitated by the present pandemic, we feel this is encouraging for future rapid research where remote methods of data collection could facilitate wider reach and minimize cost and burden of participant or researcher travel. Thirdly, the Table of Changes provides a rigorous, transparent approach for rapid data analysis to inform decisions about intervention optimisations.

### Identifying Scope for Optimisation

Research exploring intervention adaptation strategies has focused on how to identify which components or functions of an intervention can be adapted without compromising its effectiveness (33, 34). The systematic review of intervention adaptation studies conducted as part of the MRC ADAPT study suggested that identifying intervention “core components” which need to be maintained is important for defining the scope for optimisation, and proposes that these core components can be identified through the theoretical mechanisms by which the intervention is hypothesized to work (34). An understanding of the underlying theoretical mechanisms was important when deciding how to optimize Germ Defense; the intervention aimed to change behavior through increased risk perceptions, increased self-efficacy, and goal-setting, and therefore optimisations which would undermine these techniques were not acceptable. However, we were also informed by an understanding of the behavioral barriers and underlying beliefs in the target population in this context, which were summarized by the guiding principles^1^, and this was essential for informing decisions about the scope for optimisation.

### Evaluating Impact of Intervention Changes

Another important consideration in intervention adaptation is how to evaluate the impact of changes. Usage data can provide an indication of whether a change has increased engagement and, if relevant outcomes can be collected, effectiveness. For example, we found that despite a careful re-design of the front page to make it more immediately engaging and accessible, there was no change in attrition rates from this page. This might suggest that when people are motivated to use an intervention, the aesthetics and design may not have much influence on usage. This would support findings from an international RCT which showed that an interactive intervention with audio-visual features did not improve engagement or outcomes in diabetes patients over a plain-text version of the same intervention (35). Consistent with this hypothesis, Germ Defense had a much higher attrition rate from the front page when it was disseminated to the general public to reduce general colds and flu from 2016-2017 (~78% compared with the present 27%)^2^. This was in line with expectations that motivation to use Germ Defense would be higher during a pandemic situation (16), showing the importance of context and suggesting that optimisations to improve the visual appeal or simplify information may not influence usage in highly motivated populations.

The broad-brush perspective on the impact of intervention optimisations provided by intervention usage data can be complemented by qualitative research, which can enable researchers to evaluate how optimisations might influence more in-depth beliefs about the intervention and target behaviors.

### Strengths and Limitations

The priority of this project was to roll-out the intervention to as many people as possible, to maximize the potential benefits in reducing the transmission of Covid-19. This meant that implementation and dissemination were often prioritized over the research process. For example, the decision was made not to capture demographic data from users in order to allow faster ethical approval and better user engagement. We also undertook dissemination *via* multiple concurrent routes despite this making it more challenging to evaluate the effectiveness of particular dissemination strategies. However, we have shown that health research can still generate useful research findings even when interventions are evaluated in a real-world, uncontrolled setting.

Despite efforts to recruit a diverse sample for the qualitative interviews, many of the participants were highly motivated to adhere to protective behaviors during the Covid-19 pandemic, appeared to have high levels of health literacy, and felt they had the space in their home for self-isolation when required. This limited our understanding of the barriers to engaging with the intervention and adhering to target behaviors amongst people with lower levels of motivation, or with physical restrictions in their opportunity to self-isolate.

### Conclusions

Rapid optimisation methods of this kind may in future be used to help improve the speed and efficiency of optimisation and implementation of interventions, in line with calls for more rapid, pragmatic health research methods.

Adopting a rapid and iterative approach to optimizing a live intervention ensured it remained persuasive and relevant to users throughout an international crisis, in a frightening and constantly evolving context. The range of methods helped develop a detailed understanding of the possible barriers to the target behaviors, and a clear strategy for rapid stakeholder engagement was essential for informing decisions about how to address these barriers.

Changes to the design of an intervention may be less important for promoting engagement than ensuring the intervention content is motivating, credible and persuasive.

## Data Availability Statement

The original contributions presented in the study are included in the article/Supplementary Material, further inquiries can be directed to the corresponding author/s.

## Ethics Statement

The studies involving human participants were reviewed and approved by University of Southampton and University of Bath ethics committees (Registrations 56,445 dated 12/05/20, and 20-088 dated 19/03/20). The patients/participants provided their written informed consent to participate in this study.

## Author Contributions

LY, BA, and SM: conceived of rapid optimisation process. JG, LT, and KM: conducted interviews. KM, JG, LT, and SM: inputted data and decisions to Table of Changes. JD-D: analyzed usage data. KM: drafted the manuscript. All authors: developed the intervention, participated in stakeholder discussions, reviewed the manuscript, approved the content, and met authorship criteria.

## Conflict of Interest

The authors declare that the research was conducted in the absence of any commercial or financial relationships that could be construed as a potential conflict of interest.

## Footnotes

^1^Miller S, Denison-Day J, Morton K, Towler L, Groot J, Ainsworth B, et al. Applying the person based approach for rapid optimisation of a digital intervention during a pandemic: adapting Germ Defence for COVID-19. Manuscript in preparation.

^2^Miller S, Ainsworth B, Weal M, Smith P, Little P, Yardley L, et al. Process evaluations of a web-based intervention to increase handwashing during a pandemic: moving germ defence from a randomised controlled trial to public dissemination. Manuscript submitted for publication.
