# Supplementary material for: Adapting Behavioral Interventions for a Changing Public Health Context: A Worked Example of Implementing a Digital Intervention During a Global Pandemic Using Rapid Optimisation Methods
Source: Front Public Health. 2021 Apr 26;9:668197. doi: 10.3389/fpubh.2021.668197 (PMC8109268; doi:10.3389/fpubh.2021.668197)
Supplement: Supplementary file 1 [file Table_1.DOCX]

**Germ Defence Feedback and Comments**

**<*insert intervention version number and date>***

Thank you very much for providing feedback on Germ Defence. We need to keep accurate records of comments and recommendations from all stakeholders, including members of the public, clinicians and scientists. To help us do this, please use this template to provide your feedback.

**As a clinician or scientist, we would particularly value your views on the clinical content and key messages of the intervention.**

For each feedback point, please note the title of the page it refers to (this is at the top of your browser bar – e.g. germdefence.org/**intro1**’).

Just right click to ‘add new row below’ whenever you want to add more comments.

| 1. **Essential comments on clinical content**   Please note here comments that you think *must* be addressed as soon as possible for the current/next version of the intervention.  Please add a new row for each page. Just right click to ‘add new row below’ whenever you want to add more comments. | |
| --- | --- |
| Page Title (e.g. intro1)  - leave blank if comment applies to all pages) |  |
|  |  |
|  |  |

| 1. **General Comments (e.g. recommendations for additional advice, unclear language)** | |
| --- | --- |
| Page Title: |  |
